# Role of TIM-3 in ovarian cancer: the forsaken cop or a new noble

**DOI:** 10.3389/fimmu.2024.1407403

**Published:** 2024-08-13

**Authors:** Xiangyu Chang, Jinwei Miao

**Affiliations:** Department of Gynecologic Oncology, Beijing Obstetrics and Gynecology Hospital, Beijing Maternal and Child Health Care Hospital, Capital Medical University, Beijing, China

**Keywords:** TIM-3, ovarian cancer, immunity, tumor-infiltrating lymphocytes, immune microenvironment

## Abstract

T cell immunoglobulin and mucin domain-3 (TIM-3), a crucial immune checkpoint following PD1 and CTLA4, is widely found in several immune cells. Nonetheless, its performance in recent clinical trials appears disappointing. Ovarian cancer (OC), a malignant tumor with a high mortality rate in gynecology, faces significant hurdles in immunotherapy. The broad presence of TIM-3 offers a new opportunity for immunotherapy in OC. This study reviews the role of TIM-3 in OC and assesses its potential as a target for immunotherapy. The regulatory effects of TIM-3 on the immune microenvironment in OC are discussed, with a focus on preclinical studies that demonstrate TIM-3’s modulation of various immune cells in OC. Additionally, the potential therapeutic advantages and challenges of targeting TIM-3 in OC are examined.

## 1 Introduction

The receptor protein TIM-3, a member of the TIM family, is expressed in T cells, T regulatory cells, and various cells of the innate immune system. Initial investigations into TIM-3 were primarily concerned with its capacity to negatively regulate T cell function through modulating their activity (1, 2). Later, the crucial involvement of TIM-3 in natural killer (NK) cells (3), DC (4), and macrophages (5) of the innate immune system was discovered. Recent observations have highlighted the upregulation of TIM-3 expression on T cells during infections, which is closely linked to T cell exhaustion, while its elevated presence in macrophages (6) suggests a significant role in immunity against infections. Additionally, a strong correlation has been identified between TIM-3 expression and the failure to respond or development of refractoriness to therapy with anti-PD-1 monoclonal, indicating a possible mechanism for immune evasion. Therefore, the simultaneous targeting of the TIM-3 and PD-1 pathways is seen as a promising strategy. However, the outcomes of recent clinical trials have not been favorable, suggesting that success with dual PD-1/TIM-3 antibody approaches still need a long time to explore (7).

OC accounts for 2.5% of all cancer cases among women. Despite its relatively low incidence rate, it ranks as the eighth leading cause of cancer-related mortality among women (8). The introduction of platinum-based chemotherapy has only slightly improved the prognosis for patients with epithelial OC in recent decades. Refractory and relapse phenomena commonly occur in the majority of patients despite an initial favorable response to chemotherapy (9). The lack of T cell infiltration into tumors and the impaired functionality within the cold tumor category have been significant barriers to the efficacy of immune checkpoint inhibitor therapy (10), limiting the use of immunotherapies. At present, there is no recommended immunotherapy protocol for OC based on current data; nevertheless, the unique immunological environment of this cancer presents both significant challenges and opportunities for research. Efforts are being concentrated on targeting the immunosuppressive elements within the tumor microenvironment to reprogram cold tumors to acquire a thermally active phenotype, for example, through the inhibition of T regulatory cells (11). The complexity of TIM-3, characterized by its unconventional signaling, extensive-expression across various immune cell types, and interactions with multiple ligands, offers a compelling avenue for investigation in the realm of ovarian cancer immunity.

## 2 TIM-3

TIM-3, also known as hepatitis A virus cellular receptor 2 (HAVCR2), is composed of 301 amino acids encoded by HAVCR2. It is a type I membrane protein. It was first identified in 2002 (12) as a co-suppressor receptor expressed on differentiated interferon (IFN-G)-secreting CD4+ and CD8+ T cells in mice and humans, regulating type I immunity. Subsequent studies have shown that TIM-3 is expressed in a variety of immune cells, both as an activating receptor and as a suppressor receptor, lacking a clearly identifiable inhibitory signaling motif in the tail (13). In macrophages, TIM-3 acts as an inhibitory receptor that influences the polarization of pro-inflammatory phenotypes. In dendritic cells, TIM-3 promotes the clearance of apoptotic cells, enhances cross-presentation, and promotes immune tolerance. While some studies have reported that TIM-3 expression is positively correlated with NK cell function, more studies have linked TIM-3 to NK cell failure or dysfunction (14, 15). TIM-3 also interferes with innate immune signaling pathways, including TLR3/4 and NF-κB, TLR7/9, cGAS STING, and inflammasome (16). The biological function of TIM-3 is complex, and four TIM-3 ligands have been identified: GAL-9, phosphatidylserine, high mobility group protein 1 (HMGB1), and carcinoembryonic antigen-associated cell adhesion molecule 1 (CEACAM-1). TIM-3 has gained increasing attention as a marker of the CD8+ cell subgroup most prone to dysfunction. Anti TIM-3 treatment combined with anti-PD1 can improve viral and tumor clearance, while anti PD-1 therapy alone cannot achieve this effect. This also increases confidence that anti TIM-3 will achieve clinical translation.

In addition to the well-known regulatory role of immune components in TME, immune checkpoints can also function within the cancer cells through intrinsic mechanisms (17). However, the current research on the role of TIM-3 in cancer cells is primarily focused on glioblastoma. Guo et al. have shown that TIM-3 is a common signaling pathway shared by glioma cells and immune cells. Overexpression of TIM-3 enhances the invasiveness and migration of glioma cells, increases their *in vivo* tumorigenicity, indicating that TIM-3 plays a regulatory role in the malignant behavior of glioma cells (18). TIM-3 has also been shown to drive the self-renewal of human myeloid leukemia stem cells and promote leukemia progression (19). In breast cancer cells with low expression of TIM-3, overexpression of TIM-3 promotes the invasiveness of breast cancer cells, manifested by increased proliferation, migration, invasive ability, and impaired tight junction function. TIM-3 also enhances the resistance of breast cancer cells to paclitaxel (20). Kato et al. proposed in their study that the expression of TIM-3 on cancer cells in renal cancer may be a potential predictor of the efficacy of PD-1 therapy (21). Additionally, there have been studies showing that human pancreatic cancer-associated fibroblasts (CAFs) promote the expression of TIM-3, PD-1, CTLA-4, and LAG-3 on proliferating T cells (22).

## 3 TIM-3 in clinic

The TIM-3 antibody as a monotherapy does not provide significant clinical benefits, thus researchers are exploring combination therapy options. Recent clinical trials have demonstrated that the TIM-3 monoclonal antibody Sabatolimab (MBG453) exhibits some clinical efficacy against hematologic tumors in patients with MDS and AML. However, due to the Phase III STIMULUS MDS2 study failing to meet the primary endpoint of overall survival (OS), the clinical trial was unfortunately terminated (23). The early clinical data on the utilization of AZD7789, a bispecific antibody targeting anti-PD-1 and TIM-3, was presented at ESMO in 2023. Based on the disclosed abstract documents, the clinical outlook for this pipeline appears unpromising. AZD7789 demonstrated an unconfirmed partial response rate of 10% in stage IIIB-IV non-small cell lung cancer (NSCLC) patients who had previously received anti-PD-(L)1 therapy (24). Additionally, an unexpected treatment-emergent adverse event (TEAE) occurred in 82% of patients. The likelihood of achieving success with the PD-1/TIM-3 dual antibody seems quite slim. The observed clinical activity signals of the combination therapy comprising Cobolimab, an anti-TIM-3 monoclonal antibody, and Dostarlimab (Jemperli), an anti-PD-1 antibody, in heavily pretreated patients with advanced/metastatic non-small cell lung cancer (NSCLC) are highly promising. Moreover, the safety profile remains acceptable across all administered dosage levels. Nonetheless, it is important to note that the study is still ongoing (25). The current number of investigational TIM3 drugs is approximately 33; however, the majority of them are still in the preclinical stage.

## 4 Tumor immune microenvironment of OC

Ovarian cancer (OC) can create a highly immunosuppressive tumor microenvironment (TME), which is influenced by various immune cells (T cells, natural killer cells, Dendritic cells, et al.), the secretion of ligands (Cytokines et al.) and non-immune characteristics (Cancer-related fibroblasts et al.) (26). These components collectively play a crucial role in cancer progression, peritoneal spread, and chemotherapy resistance, making them potential therapeutic targets. Among the key factors contributing to this unique immune environment, OC TME is heavily infiltrated by regulatory T cells (TREGs), M2-polarized macrophages, and other myeloid progenitor cells that promote immune evasion and contribute to carcinogenic progression (27). Soluble factors such as IL10 and VEGF are also important components of TME, which contribute to the migration and proliferation of cancer cells.

A tumor with a large number of tumor-specific CD8+T cell responses is referred to as a “hot tumor”, OC is typically considered a cold tumor or warm tumor. However, with the development of single-cell sequencing technology, researchers have found that HGSOC tumors contain significant levels of TIL, Treg, and CD8 exhausted T cells that are significantly enriched in the ovarian lesion (primary tumor), but the tumor is still progressing malignantly (28–30) T cell exhaustion is a regulatory mechanism that limits the activity and effector function of T cells under chronic antigen stimulation. In the process, Antigen-specific CD8+ T cells show a decreased cytotoxic activity, a gradual loss of the production of cytokines such as IL-2, TNFα, and IFN-γ, but still produce granzyme B, thus possessing certain immune regulatory ability, but not enough to curb disease progression (31). TIM-3 can serve as a marker for T cell exhaustion (32). Studies have shown that PD-1(+) TIM3 (+) CD8 TIL are failing T cells with low tumor immune function, and functional PD-1(+) TIM-3(+) CD8 TILs have been found in several cancers (33–37). In Renal Cell Carcinoma, the percentage of tumor-infiltrating CD8 T cells co-expressing PD-1 and TIM-3 correlates with an aggressive phenotype at diagnosis and a larger tumor size (33). In gastric cancer, CD8+T cells expressing PD-1 and TIM-3 produce significantly lower interferon-gamma than CD8+T cells expressing PD-1 (+) and TIM-3 (-) (34). In the CRC TME, the level of CD4+FoxP3+Helios+T cells, which represent highly immunosuppressive Tregs, significantly increases. CTLA-4, TIM-3, and LAG-3 are mainly co-expressed on FoxP3+Helios+Tregs in the TME, suggesting that Tregs may hinder the response of colorectal cancer patients to IC blockade (35). Study showed that the perforin+ percentage of PD-1(+) TIM-3 (+) CD8 TIL in ovarian cancer was lower than that of PD-1(-) TIM-3(-) and PD-1(+) TIM-3(-) CD8TIL; the granzyme B+ percentage of PD-1+TIM-3+CD8TIL in OC was also lower than that of PD-1(-) TIM-3(-) and PD-1(+) TIM-3(-) CD8 TIL. The analysis was conducted using TOX expression as an indicator of exhaustion. PD-1(+) TIM3 (+) CD8 TIL was higher than that of PD-1(-) TIM-3 (-) and PD-1(+) TIM-3 (-) CD8 TIL. Although, PD-1(+) TIM-3(+)CD8 TILs in OC show sustained IFN-γ production and proliferation potential, cytotoxic capacity is impaired, which, taken together, suggests that PD-1(+) TIM-3(+)CD8 TILs in OC, although heterogeneous, are in a state of exhaustion (38). The high expression level of TIM-3 in these cells suggests that TIM-3 plays a key regulatory role of in the immunosuppressive environment of OC. TILs with impaired function may be identified by a reduction in T cells, decreased cytokine production and proliferation, diminished cytotoxicity, and an upregulation of immune checkpoints such as PD-1 and TIM-3. More importantly, TIM-3 can also serve as a target for therapeutic intervention to restore T cell function. By targeting TIM-3 with drugs, T cell function can be restored, allowing them to continue fighting pathogens or tumor cell (32).

**Figure 1:**
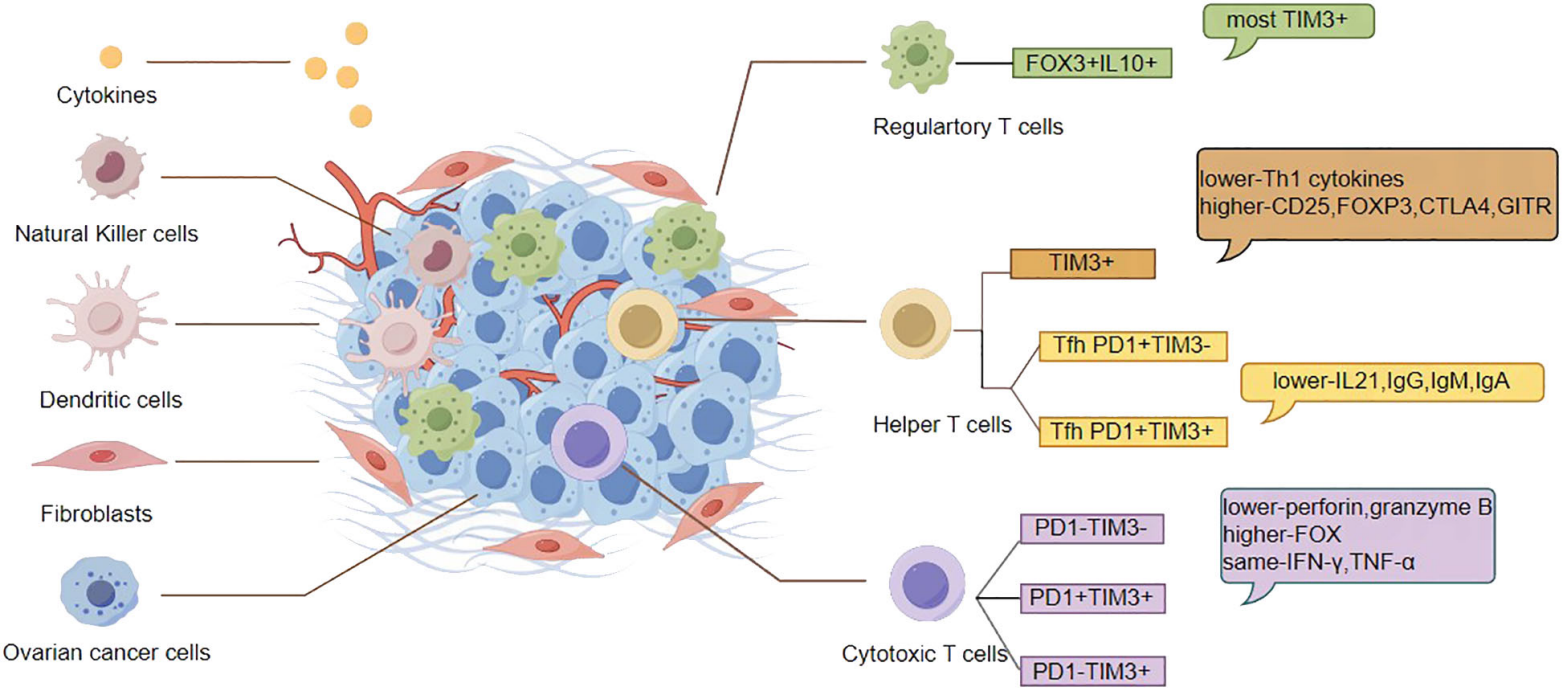
Characteristic T cells associated with TIM-3 in the TME of OC. Regulatory T cells: Tregs contain significantly higher frequencies of IL-10+ cells, most of which belong to TIM-3+ cells. Helper T cells:TIM-3(+) CD4 T cells in TME produce lower levels of Th1 cytokines and express higher levels of CD25, Foxp3, CTLA-4, and GITR, The PD-1+ Tfh cell population can be further segmented based on TIM-3 expression, with TIM-3(+)PD-1(+)Tfh cells showing slightly lower secretion of IL-21 and proliferation than TIM-3(-)PD-1(+) counterparts, as well as a significantly weaker ability to enhance IgM, IgG, and IgA response. Cytotoxic T cells: The perforin+ and granzyme B+ percentage of PD-1(+) TIM-3 (+) CD8 TIL in OC was lower than that of PD-1(-) TIM-3(-) and PD-1(+) TIM-3(-) CD8 TIL; TOX expression was higher than that of PD-1(-) TIM-3 (-) and PD-1(+) TIM-3 (-) CD8 TIL; PD-1(+) TIM-3(+)CD8 TILs in OC show sustained IFN-γ and TNF -α production.

In summary, although the ovarian cancer TME is extremely complex and heterogeneous targeting this environment remains highly relevant and promising (39), however we face multiple challenges thus it is critical to provide the most effective combination strategies for immunosuppression. TIL, which is the most important part of OC TME, always deserves attention, and T cells are the key components of TIL. Currently, immunotherapy is mostly focused on T cells, and research on TIM-3 on T cells is increasing. TIM-3 has the potential to become a new star in improving immunotherapy for OC. In the following, we will mainly review the potential applications of TIM-3 on T cells in OC (**Figure 1**).

## 5 The role of TIM-3 in T cells of OC

### 5.1 Cytotoxic T cells and helper T cells

CD8+ T cells can directly kill virus-infected somatic cells as well as tumor cells. CD4+ T cells (also referred to as helper T cells) play a pivotal role in modulating both immune and non-immune cellular responses by orchestrating the production of cytokines. Certain T cells, especially CD8+ T cells, may become dysfunctional in chronic inflammatory or tumor microenvironments. TILs in ovarian cancer specimens are a powerful biomarker that can predict the overall survival rate of women with this disease (40). TIM-3 is widely expressed by immune cells in TME and has been identified as a poor prognostic biomarker in over 9,000 patients with solid tumors in the Cancer Genome Atlas (41), TIM-3 was expressed in these cells, and it affects the prognosis of patients with OC. In analyses of clinical samples, Wu et al. observed a marked increase in TIM-3 expression on both CD4+ and CD8+ T cells in peripheral blood of OC patients compared to a control group. Notably, the proportion of TIM-3+CD4+ T cells was higher in patients with recurrent OC than in those with primary OC (p=0.013). TIM-3 expression was also more pronounced in CD4+ and CD8+ T cells from Patients exhibiting a higher tumor grade (G3)compared to those with a lower tumor grade (p=0.010 and p=0.042) (42). Furthermore, Li et al. found a strong correlation between high levels of PD-L1 and the density of PD-L1+ cells in the tumor microenvironment of high-grade serous carcinoma (HGSC), resulting in enhanced clinical outcomes through Th1 polarization and cytotoxic orientation. However, the independent prognostic value of PD-1 and TIM-3 co-expression by CD8+ CTLs points to functional exhaustion and signifies TIM-3’s dominant role in the immunosuppression observed in therapy-naive HGSC patients. These data suggest that PD-L1 and TIM-3 may be prognostic biomarkers of active and inhibitory immune responses against HGSC (43).

In a broader study, Zheng et al. (44) characterized TIM-3(+) CD4 T cells in specimens from patients diagnosed with various cancers, including OC. The study revealed that the frequency of TIM-3(+) CD4 T cells was significantly higher in tumor tissues than in non-tumor invasive lymphocytes from peripheral blood. These TIM-3(+) CD4 T cells in tumor tissues produce lower levels of Th1 cytokines and express higher levels of CD25, Foxp3, CTLA-4, and GITR. Additionally, TIM-3(+) Foxp3 (+) CD4 T cells are preferentially distributed within the tumor nest rather than the peri-tumor matrix, which may contribute to the immunosuppressive tumor microenvironment. Zhang et al. (45) demonstrated that the combined use of anti-TIM-3/CD137 monoclonal antibodies effectively inhibited the growth of ID8 OC cells and significantly increased long-term survival in mice, an effect not observed with the individual antibodies. Targeted inhibition of TIM-3 is expected to revitalize T cell activity. However, more in-depth research on TIM3 mechanism and clinical studies in ovarian cancer are needed.

T follicular helper cells (Tfh) are a specialized subset of CD4+ T cells. They play a critical role in for B cell assistance in germinal centers and peripheral blood, exhibit high levels of PD-1 (46–48). A severe depletion in CD8 T cells is characterized by the co-expression of TIM-3 and PD-1 (49–51). It raises the question: Is TIM-3 also expressed on PD-1 high Tfh cells? Comparisons between PD-1+ Tfh cells from NC control and OC patients showed that PD-1+ Tfh cells in OC patients secreted higher levels of IL-21 and IL-10 than those from NC controls, indicating that PD-1 presence on Tfh cells reflects increased activity rather than exhaustion. The PD-1+ Tfh cell population can be further segmented based on TIM-3 expression, with TIM-3−PD-1+Tfh cells showing slightly higher secretion of IL-21 and proliferation than TIM-3+PD-1+ counterparts, as well as a significantly greater ability to enhance IgM, IgG, and IgA responses. Investigating the role of TIM-3+PD-1+Tfh cells in healthy subjects is an important direction for future research, although technical challenges due to the low frequency of Tfh-TIM-3+ cells in the peripheral blood of healthy individuals may require animal studies for thorough examination (52).

### 5.2 Regulatory T cells

Regulatory T cells (Tregs) are a subset of CD4+T cells that typically express the transcription factor forkhead box protein P3 (FOXP3). By directly inhibiting suppressive cytokines such as IL-10, IL-35, and transforming growth factor beta (TGF-beta), as well as indirect inhibitory mechanisms such as the subversion of dendritic cells towards more immune regulatory phenotypes, the depletion of local IL-2 (a key T cell growth factor), a suppressive tumor microenvironment is established. Tregs represents a promising therapeutic approach to enhance outcomes in transplantation and autoimmunity (53).An increased subset of CD8+Treg cells has been noted in patients with OC compared to those with benign ovarian tumors and healthy individuals (54, 55). Cai et al. (56) showed that the Tregs infiltrating ovarian tumors exhibited a higher degree of immunosuppression compared to their counterparts in peripheral blood. Treg cells secrete IL-10, which mediates immune suppression (57), including the inhibition of pro-inflammatory cytokine production by CD8+T cells. This study demonstrates that compared to PBMC Tregs under non-stimulating conditions, TIL Tregs contain significantly higher frequencies of IL-10+ cells, most of which belong to TIM3+ cells. This suggests that the suppression of CD8+T cell activity and IL-10 production mediated by TIL Tregs are likely dependent on TIM-3. **Table 1** shows the expression of TIM-3 and its relationship to the different microenvironments in OC.

**Table 1:** TIM-3 expression on tumor-associated immune cells and study observations related to expression in OC.

References	Lymphocyte Subset	Major Types of Cells	Observations
(38)	TILs	PD-1+ TIM-3+ CD8+T	Potential effects on cytokine production, proliferation, and cytotoxicity
(42)	PBLs	TIM-3+CD4+T, TIM-3+CD8+T	Elevated TIM-3 expression in both CD4+ and CD8+ T cells in cases with advanced staging (III/IV) than those with stage I and II
(43)	TILs	PD-1+TIM-3+CD8+ T	Associated with a poor prognosis of HGSC
(44)	TILs	TIM-3+CD4+T	TIM-3+CD4 T cells may represent functional regulatory T cells that contribute to the formation of immunosuppressive tumor microenvironments.
(45)	TILs	Anti-TIM -3/CD137	Combined TIM3 blockade and CD137 activation affords the longterm protection ina murine model of ovarian cancer
(52)	TILs,PBLs	TIM-3+ PD-1+ Tfh	The level of IL-21 secretion and proliferation of TIM-3+ PD-1+ Tfh cells were decreased. The ability of TIM-3+ PD-1+ Tfh cells to induce the secretion of IgM, IgG and IgA by B cells was significantly impaired. The frequency of PD-1+ Tfh cells and TIM-3+ PD-1+ Tfh cells in TILs was significantly higher than in PILs.
(56)	TILs,PBLs	Treg	Expression of TIM3 on TIL Tregs was directly correlated with tumor size. Ovarian TIL Treg cells were more immunosuppressive than peripheral blood counterparts in a TIM3-dependent fashion.
(58)	MALs,TILs,PBLs	Vδ1 T	TIGIT and TIM-3 are highly expressed in MALs and PBLs. The Vδ1 T cell population has a high prevalence in primary tumors in patients with MALs and OvCA.

TILs, tumor-infiltrating lymphocytes; PBLs, peripheral blood lymphocytes; MALs, malignant ascites lymphocytes.

### 5.3 γδ T cells

γδ T cells, characterized by their γδ TCR, play a pivotal role in the innate immune response by mounting rapid reactions against infections and tissue damage. These cells can also exhibit immunosuppressive or tumor-promoting effects, especially through IL-17 secretion (59). γδT cells are categorized into Vδ1, Vδ2, and Vδ3 T cells. Fiedler’s research indicated an increase in TIM-3 expression on Vδ1 T cells, aligning with Xioma et al.’s finding of elevated TIM-3+ cell frequency within the total γδ T cell population in colorectal cancer. Additionally, anti-TIM-3 therapy was shown to boost the *in vitro* cytotoxicity of Vδ2 T cells in colorectal cancer, although similar findings were not reported in OC (58, 60).

## 6 Role of TIM-3 in the resistance to immunotherapy of OC

The most difficult key point of OC immunotherapy is that tumor-inhibiting immune cells and immune cells that promote tumor growth often coexist in the same location within the TME of OC (61). TIM-3, as a factor widely expressed in both types of cells, presents an opportunity to overcome immunotherapy resistance to OC.

Current immunotherapy for OC can be divided into three categories: immune checkpoint inhibitors, therapeutic vaccines, and adoptive cellular immunotherapy (mainly CAT). PD-1/PD-L1 inhibitors are only effective in a small percentage of OC patients compared to other tumors. The expression of multiple immune checkpoints in T cell subsets may be a key reason for resistance to immunotherapy in OC (62). Double or triple immune checkpoint blockade may be beneficial for OC patients and may help overcome immune resistance (63, 64), suggesting that an immune checkpoint inhibitor combined with TIM-3 is worth looking forward to, but there are currently no reliable clinical trial results. OC vaccine treatment has been investigated in various clinical trials. So far, tumor vaccines used alone have not shown good responses in OC, and the improvement of their effectiveness remains an important issue (65). At present, there is little research on immunotherapeutic vaccines targeting TIM-3.

CAR-T cells are T cells that have been genetically engineered to express chimeric receptors that target specific antigens. They are equipped with chimeric antigen receptors (CARs) that enable T cells to recognize and destroy cancer cells expressing corresponding antigens to achieve the goal of treating cancer. One of the most challenging limitations of CAR-T cell therapy is the tumor’s resistance to targeted CARs of a single antigen. Tumor cells lacking death receptor molecules are prone to T cell exhaustion when subjected to prolonged stimulation by CAR-T cells. Currently, the role of immune checkpoint blockade in overcoming T cell exhaustion has been applied to CAR-T cells (66). Immunotherapy that combines CAR-T cells and checkpoint blocking is considered the next immunotherapy frontier (67). In a study conducted by Jafarzadeh L, a specific shRNA targeting the human TIM-3 gene was designed, and it was co-inserted into a lentiviral vector with the MSLN-CAR-T transgenic construct. The results showed that knocking down TIM-3 significantly reduced its expression in the MSLN-CAR-T cells, significantly improved the cytotoxic function, cytokine production, and proliferation ability of the MSLN-CAR-T cells. Overall, targeted knockdown of TIM-3 allows tumor-infiltrating CAR-T cells to proliferate and function effectively, thereby alleviating TIM-3-mediated immune suppression (68).

CAR-T therapy has demonstrated lasting clinical responses in various cancers, with tumors expressing higher levels of mesothelin in advanced stages (III and IV) and in high-grade EOC. Anti-mesothelin CAR T cells (mesoCAR T cells) are under clinical trials for several cancer types, including EOC (69–72). High expression of inhibitory receptors such as PD1, TIM-3, and A2aR has been observed during the manufacturing of meso-CAR T cells for advanced human epithelial OC (73), suggesting that optimizing CAR T cell production protocols might overcome the need for pre-activation of T cells. This suggests that a CAT-T regimen modified for TIM-3 has a high potential to be effective in OC.

## 7 Conclusion

TIM-3 plays a crucial role in regulating the function of myeloid cells (including macrophages, dendritic cells, neutrophils and mast cells) (15, 74, 75). Although the current clinical trial results of TIM-3 are not satisfactory focused on TIM-3 (23), the role of TIM-3 in OC is still worthy of further exploration, and the current research lacks thorpness. There is no convincing evidence of the significant application effectiveness of TIM-3. Although TIM-3 may not be as important as PD-1 and other immune checkpoints, its potential contribution, such as an imaging marker for PET/CT, maintains its relevance (32, 76, 77).

The potential application of TIM-3 in ovarian cancer is mainly focused on two aspects. On the one hand, the abnormal expression of TIM-3 in ovarian cancer suggests its potential role as a biomarker. On the other hand, blocking TIM-3 can significantly reverse Treg-mediated CD8 inhibition, so TIM-3 has the potential to be a therapeutic target for overcoming immunotherapy resistance to OC (78). Therefore, TIM-3 is expected to become a “New Noble” of future research in the context of OC.
